# Profanity as a Self-Defense Mechanism and an Outlet for Emotional Catharsis in Stress, Anxiety, and Depression

**DOI:** 10.1155/2023/8821517

**Published:** 2023-05-03

**Authors:** Waqar Husain, Samia Wasif, Insha Fatima

**Affiliations:** Department of Humanities, COMSATS University, Park Road, Islamabad, Pakistan

## Abstract

**Background:**

Swearing is an increasing trend among men and women worldwide. Earlier studies on the positive aspects of profanity mostly relate to pain management and the release of negative emotions. The uniqueness of the current study is its analysis for a possible constructive role of profanity in stress, anxiety, and depression.

**Method:**

The current survey involved 253 conveniently selected participants from Pakistan. The study analyzed the role of profanity in connection to stress, anxiety, and depression. Profanity Scale and the Urdu version of Depression, Anxiety, and Stress Scale were used along with a structured interview schedule. Descriptive statistics, Pearson's correlation coefficient, and *t*-test were implied to obtain results.

**Results:**

The study revealed that the usage of profane language had significantly inverse correlations with stress (*r* = −0.250; *p* < 0.01), anxiety (*r* = −0.161; *p* < 0.05), and depression (*r* = −0.182; *p* < 0.01). Higher profaners also revealed significantly lower levels of depression (M = 29.91, SD = 10.80 vs. M = 33.48, SD = 10.40; *p* = 0.009; Cohen's *d* = 0.338) and stress (M = 30.83, SD = 11.41 vs. M = 35.16, SD = 11.31; *p* = 0.003; Cohen's *d* = 0.381) as compared to lower profaners. Profanity had no significant correlations with age (*r* = 0.031; *p* > 0.05) and education (*r* = 0.016; *p* > 0.05). Men projected significantly higher levels of profanity as compared to women.

**Conclusion:**

The current study viewed profanity similar to the self-defense mechanisms and emphasized on its cathartic role in stress, anxiety, and depression.

## 1. Introduction

### 1.1. Self-Defense Mechanisms

Humans are programmed to seek pleasure and avoid pain [1]. They want to repeat pleasurable and emotionally satisfying situations [2]. Based on the same pleasure principle, Sigmund Freud proposed a concept of the unconscious related self-defense mechanisms which are considered as the counter forces that activate in reaction to anxiety and negative feelings [3–5]. Defense mechanisms function at four hierarchical levels, i.e., from the least to the most mature [6], and include the psychotic defenses (delusional projection, psychotic denial, and psychotic distortion), the immature defenses (acting out, autistic fantasy, dissociation, hypochondriasis, passive aggression, and projection), the intermediate defenses (displacement, intellectualization, reaction formation, and repression), and the mature defenses (altruism, anticipation, humor, sublimation, and suppression). Repression, for example, is a flight from pain [7] in which a person tends to forget the painful and traumatic experiences of life [8, 9]. In denial, a person may refuse or deny a situation to exist [10]. Projection makes a person to attribute his unwanted thoughts and feelings to another person [11]. Displacement is to redirect an impulse of aggression to a comparatively easier and less powerful target. In regression, a person exhibits an unsuitable behavior and fantasy to avoid blame from self and others [8, 12]. Sublimation is also a self-defense mechanism in which a person tries to find satisfying ways to displace the unacceptable emotions into behaviors which are constructive and socially acceptable [8]. Sublimation is considered the most mature self-defense mechanism as it enables the person to satisfy the intolerable urges through socially acceptable and satisfying ways [12–14]. Sublimation functions as a healthy, creative, socially acceptable, and useful resolution of inner conflicts between original urges and hindering forces [15]. Defense mechanisms are unconscious, beyond the knowledge of the user and appearing as odd behaviors to the observers, dynamic and reversable, adaptive and creative, and the major means of mitigating the distressing effects of both intense emotion and cognitive dissonance [6].

The concept of defense mechanisms is widely accepted [12, 16] as it is visible in everyday behavior and emotions [17] of both men and women [18]. Defense mechanisms are regarded significant in human functioning [6]. Defense mechanisms alter perception [19] unconsciously [3] without involving the cognitive mechanisms for coping [3, 20]. Defense mechanism protects a person from the threatening and harmful biological changes, severe anxiety, depression, personality disorders, losing self-esteem, feeling ashamed, falling in cognitive conflicts, accepting failures, irresponsibility, self-centeredness, maladaptive functioning, eating disorders, somatic problems, and being affected from several negative thoughts [3, 12, 16, 18, 21–41].

### 1.2. Emotional Catharsis

Self-defense mechanisms are primarily oriented toward emotional catharsis. Emotional catharsis means the intense expression of emotions [42], the discharge of repressed or immediate emotions to release tension [43], and releasing the hostile [44] or traumatic [45, 46] feelings from mind. Studies have established the positive effects of emotional catharsis in releasing psychological distress during the psychotherapeutic procedures or in the daily life conditions [46–49]. Achieving emotional catharsis during psychotherapy is seen as an important cause of psychotherapeutic success [46, 50–52] as it helps in building rapport with the clients which strengthens the foundations of the psychotherapeutic relationship. Studies have also established that crying [53, 54], listening to music [55, 56], engaging in martial arts [57], and aggressive activities [58] such as playing violent video games [59, 60] help individuals to cope with life stressors and negative emotions.

### 1.3. Profanity

Profanity refers to offensive [61] and aggressive [62, 63] taboo words [64] related to body parts, bodily functions, sex, religion [65], and language based on positive or negative emotions [61, 66] and is subject to the subjective interpretation of the receiver [67] and the context in which profane language is used [68]. Profanity can be an unfiltered and abrupt expression of emotions [69] and a planned insult of an object, place, or a person [70]. Profanity is a fundamental part of language [71] and has its own grammatical structure [72]. Sociolinguistics correlates language and society ([73]; J. [74]) and tries to find out the relationship of profane language with gender, age, ethnicity, social class, context [75], religion, and political differences [76]. Research has established the biological effects of hearing or reading profane language such as the faster heartbeat, changes in respiration, blushing, autonomic arousal, increased galvanic skin response, and loss of bowel or bladder function (A. F. [77]). People with lesser agreeableness, lesser conscientiousness, higher extraversion, higher hostility, and antisocial personality traits swear more than their counterparts [78, 79]. People with sexual anxiety and sexual repression swear less [70].

Using profane language was punishable in 15^th^ century (Teresa E. [65, 80]). Profane language, in the current times, has been losing its power [81] and has becoming more common [82]. Profanity is more tolerable in private gatherings as compared to formal meetings [64, 83] and within the same gender groups as compared with groups involving mixed genders [61]. The objectives of using profane language include social bonding, handling emotional and physical pain, emotional catharsis, expressing power and control, establishing dominant-submissive relationships, confronting authorities, labeling others, conveying aggression, and humiliating others [64, 66, 67, 84–90]. Profanity is also used for humor and comedy [91–93] and to stimulate sexual excitement (Teresa E. [80]). The profane words of one's native language contain more emotional strength [94].

Profanity can be used for both constructive and destructive purposes [95]. Swearing is a natural response to release stress [82, 96], to avoid pain [97], and to avoid severe mental consequences [66, 98]. Profane words tend to express deep emotional feelings [99, 100]. Words like “shit,” “fuck,” “bullshit” can express both positive and negative feelings [101]. Complimentary profane words such as “fucking brilliant” are also considered polite under certain circumstances [102]. Profanity can lead to tighter emotional and social bonds and a harmonious environment [64, 70, 83]. It also indicates the closeness of speaker and swearer and whether they consider each other belonging to a shared intimate or social group [64, 103, 104]. The use of profanity within a group is also the symbol of acceptance, inclusiveness, and the improvement of relationships by breaking social taboos together [102]. Swearing has an established positive effect on social interactions regarding social cohesion, comfort, and familiarity [70]. A study suggested that managers should adopt a rather permissive leadership style with respect to swearing [82]. Similarly, groups of adolescents use swearing as a sign of solidarity [63]. Profanity has also been positively correlated with honesty (G. [105]).

### 1.4. Rationale of the Current Study

Swearing and cursing can serve as an unconscious and mature self-defense mechanism to reduce the adverse effects of daily stressors. The earlier studies have also emphasized on the similar roles of profanity in releasing stress [82] and avoiding the fear of pain [90]. The literature, however, lacked studies focusing on the reduction of stress, anxiety, and depression through profanity. The current study, therefore, identified this knowledge gap and intended to analyze the role of profanity in connection to stress, anxiety, and depression. The study perceived profanity similar to self-defense mechanisms and regarded it as an outlet for emotional catharsis.

## 2. Method

### 2.1. Participants

The study involved 253 conveniently selected adult participants from Pakistan. These include both men (*n* = 98) and women (*n* = 155).

### 2.2. Instruments

As the mother tongue of the participants was Urdu, the instruments used in the study were also in Urdu for getting more reliable results. Profanity Scale [106] is a self-respondent scale in Urdu which intends to measure the frequency of using profane language in daily conversation. The scale comprises of 62 items and records responses through a 5-point Likert scale ranging from strongly disagree to strongly agree. The developers reported strong validity (communalities for all the items ranged between 0.58 and 0.87), and excellent reliability (*α* = 0.967; item-total and item-scale correlations were highly significant at 0.001 level) for the scale. The scale reports 12 aspects involved in profanity, i.e., happiness, aggression, sexual frustration, revenge, attention seeking, thrill seeking, emotional satisfaction, avoiding physical fights, conversational habit, dominance, emotional immaturity, and power seeking. The study also used the Urdu translated version [107] of the Depression, Anxiety, and Stress Scale [108]. The scale is based on the tripartite model of depression and anxiety [109] which focuses on both the common and uncommon features of depression and anxiety. The earlier studies have supported the usefulness of the tripartite model of depression and anxiety [110, 111] and the clinical reliability of the Depression, Anxiety, and Stress Scale [112–115]. The items of the scale are based on the prime features of depression (dysphoria, hopelessness, devaluation of life, self-deprecation, lack of interest, anhedonia, and inertia), anxiety (autonomic arousal, skeletal musculature effects, situational anxiety, and subjective experience of anxious effect), and stress (difficulty relaxing, nervous arousal, easy agitation, irritability, over-reaction, and impatience). The Urdu version is also a self-respondent scale comprising of 42 items. It uses a 4-point Likert scale to gather responses and asks the respondents to report the possible symptoms of stress, anxiety, and depression present in the last week. The developers reported strong validity (communalities for all the items ranged between 0.71 and 0.96) and excellent reliability (*α* = 0.910; item-total and item-scale correlations were highly significant at 0.001 level) for the scale. Both these scales were found excellently reliable in the current study too (Table 1). A demographic information schedule was also used to obtain data about gender, age, education, and contact details of the participants. A structured interview schedule was also used with the 30 selected interviewees to obtain their views on the connections of profanity with psychological distress. The questions of the interview reflected upon the instances where the interviewees successfully managed their distress through profanity.

### 2.3. Procedure

The researchers approached the participants of the study individually while visiting different hospitals, clinics, educational institutions, and public offices. The participants were informed about the purpose of the study, and their consent to participate in the study was appropriately taken. They were assured for the confidentiality of the data and were thanked for their participation. The study was approved by the Ethics Review Committee of the Department of Humanities, COMSATS University Islamabad, Pakistan. All the procedures performed in this study were in accordance with the 1964 Helsinki Declaration and its later amendments.

### 2.4. Analysis

The data were recorded in the Statistical Package for Social Sciences (version 23). Descriptive statistics were calculated to measure the accuracy of the data and to quantify the findings of the interviews. This involved measuring item range, skewness, and kurtosis. Pearson's correlation coefficient was applied to measure the correlations of profanity with stress, anxiety, depression, age, and education. *t*-test was used to measure the gender-based differences in using profane language, stress, anxiety, and depression. The probability level was 95%.

## 3. Results

### 3.1. Reliability of the Instruments

The findings revealed excellent reliability for profanity (*α* = 0.983), depression (*α* = 0.917), anxiety (*α* = 0.906), and stress (*α* = 0.923) scales (Table 1).

### 3.2. Levels of Profanity, Depression, Anxiety, and Stress

The level of profanity among the participants was 41.88% (Table 1) as calculated through the mean score. The levels of depression (57.30%), anxiety (50.21%), and stress (59.77%) were also recorded through the mean scores of the participants (Table 1).

### 3.3. Correlations of Profanity with Stress, Anxiety, and Depression

The usage of profane language had significantly inverse correlations (Table 2) with stress (*r* = −0.250; *p* < 0.01), anxiety (*r* = −0.161; *p* < 0.05), and depression (*r* = −0.182; *p* < 0.01), as predicted.

### 3.4. Correlations with Age and Education

Profanity had no significant correlations with age and education (Table 2). Stress (*r* = −0.205; *p* < 0.01), anxiety (*r* = −0.129; *p* < 0.05), and depression (*r* = −0.192; *p* < 0.01) had significantly inverse correlations with age (Table 2). Stress (*r* = −0.138; *p* < 0.05) and depression (*r* = −0.136; *p* < 0.05) also had significantly inverse correlations with education (Table 2).

### 3.5. Comparisons between Lower and Higher Profaners

The inverse correlations of profanity with depression, anxiety, and stress were further tested by making two groups out of the respondents. These groups were based on the mean score on profanity (Table 1; M = 130), i.e., lower profaners whose score on profanity was till 130 and higher profaners whose score on profanity was above 130. Higher profaners projected significantly lower levels of depression (Table 3; M = 29.91, SD = 10.80 vs. M = 33.48, SD = 10.40; *p* = 0.009; Cohen's *d* = 0.338) and stress (Table 3; M = 30.83, SD = 11.41 vs. M = 35.16, SD = 11.31; *p* = 0.003; Cohen's *d* = 0.381) as compared to the lower profaners. The differences between higher and lower profaners for anxiety were not statistically significant (Table 3; M = 26.48, SD = 8.95 vs. M = 29.17, SD = 10.49; *p* = 0.036) but reflected a trend of reduced anxiety for higher profaners.

### 3.6. Differences in Men and Women

Men projected significantly higher levels of profanity as compared to women (Table 4; M = 143.235, SD = 58.392 vs. M = 121.374, SD = 57.638; *p* = 0.004; Cohen's *d* = 0.377). Women projected significantly higher levels of stress as compared to men (Table 4; M = 34.761, SD = 11.413 vs. M = 31.439, SD = 11.467; *p* = 0.025; Cohen's *d* = 0.291). Men and women did not project significant differences in anxiety and depression (Table 4).

## 4. Discussion

The uniqueness of the current study is its analysis for a possible correlation of profanity with stress, anxiety, and depression. The earlier literature lacked findings on this specific connection. The experiences of stress, anxiety, and depression are quite normal in our daily lives. However, the excessive, unrealistic, and abnormal behaviors resulting from stress, anxiety, and depression may lead us to the clinically significant levels that may require treatment. Psychological distress, simply labeled as stress, is a change in one's emotional state caused by the psychosocial stressors in life [116]. Anxiety refers to the unrealistic and excessive worry about the future [117]. It is considered the most common complaint of the outdoor patients in hospitals [118], a chronic illness [119, 120], and is highly prevalent worldwide [121]. Anxiety is positively correlated with several other mental disorders [122] and chronic physical conditions ([123]; A. C. [124, 125]). Depression is an umbrella term used for the subcategories of depressive and bipolar disorders. Depression is a global challenge (Correction [126, 127]) and the most frequent mental disorder worldwide [128–132]. Depression may also lead to diabetes, cardiovascular disease [133], premature deaths [134], suicide [135, 136], etc. Studies also reveal that people, in many parts of the world, lack sufficient knowledge on mental health [137], still stigmatize mental disorders [138], and stay reluctant to seek professional psychological help [139]. They are more inclined toward self-made strategies to deal with psychological problems [140]. Profanity, in such an environment, can be viewed as a cultural alternative to manage stress, anxiety, and depression. Expression of negative emotions has been regarded as the main purpose of swearing [61, 62, 68, 69].

The current study has found significantly inverse correlations of profanity with stress, anxiety, and depression. The findings of the current study also revealed significantly lower levels of depression and stress among respondents who scored higher on profanity. These significant differences further established the positive effects of profanity in depression and stress. Profanity is a form of emotional expressivity which refers to the positive or negative expressions of emotions through verbal or nonverbal communication [141]. Emotional expressivity is regarded helpful for a better psychosocial wellbeing [142, 143]. Suppressing or repressing emotions, on the other hand, has been seen negatively with regard to mental health [21, 37]. Emotional suppression and repression have been positively linked up with depression [32, 34, 35], anxiety [39], personality disorders [18, 30, 40], low self-esteem [34], irresponsibility, self-centeredness [16, 26–28, 38, 41], maladaptive functioning [3], eating disorders [25], somatic problems [29], etc. Cognitive hyperactivity is an integral part of both anxiety [144] and depression [145]. Although the earlier literature is silent in reporting the specific connections of profanity with stress, anxiety, and depression, several relevant findings do support the notion that profanity may help in reducing stress, anxiety, and depression, the same way as it stays helpful in reducing physical pain [87, 90, 97, 146–151] and the effects of negative emotions [62]. Profanity has an established cathartic effect [62, 66]. It represents the intensity of the mental state of a person [72] and discharges negative emotions in a cathartic fashion [62]. Cathartic swearing has been considered to release negative emotions [104]. Cathartic swearing can be done with or without an audience [104] and is neither polite nor rude [64, 104, 152]. Some studies have projected that the use of profane language by therapists during the therapeutic session resulted in the negative evaluation of the therapists by the clients [67]. The clients perceived the therapists using profane language as dishonest, incapable, dissatisfying [153], untrustworthy (M. [154]), physically unattractive [155], disrespectful, unprofessional, and insensitive to the needs of the clients [156]. Some other studies, however, have portrayed the positive effects of using profane language during the therapeutic session ([157]; M. J. [100, 153, 156, 158–160]) such as to create an impression that the therapist is understanding and relatable to the clients [160], to oppose the prevailing culture [161], to make the conversation more effective (G. [105, 160]), and to be more convincing [67] like the public speakers who use profane language in their speeches that were found to be more effective (M. D. [162]), more persuasive and intense [93], and more trustworthy for the listeners [62]. A study required the respondents to listen to the recorded conversation between therapists and clients and found that the respondents, upon the use of profane words by clients, found the therapist involved as more expert, attractive, and more trustworthy [156].

The additional findings of the current study revealed significantly higher levels of profanity among men as compared to women. These findings are in line with the earlier literature in this regard. Women have been regarded ethically stronger than men [163]. Studies have reported higher trends of swearing among men and regarded men as angrier than women because their anger is socially more acceptable [61, 66, 164–173]. Some other studies, however, did not find any significant differences in this regard and concluded that men and women swear equally ([62]; D. I. [174–177]). A study has rather claimed differently by reporting that women swear more than men [95]. Swearing, however, has been more socially acceptable for men as compared to women [91]. The current study also analyzed the relationship of profanity with age and education and found no significant correlations in this regard.

### 4.1. Limitations

The study could have been more fruitful if the experimental design would also have been incorporated in addition to the traditional data collection through scales and interviews. The study would have been more accurate if the sample would have been more diversified by including the elderly and uneducated participants as well.

## 5. Conclusion

The current study contributed to the existing body of scientific knowledge by correlating profanity with stress, anxiety, and depression. The study viewed the positive effects of profanity similar to the role of self-defense mechanisms in releasing psychological distress. Furthering the existing studies on pain and stress management, the current study highlighted the cathartic role of profanity in stress, anxiety, and depression as well.

### 5.1. Suggestions for Future Researchers

Researchers are advised to further explore the positive aspects of profanity in the management of other significant mental disorders. Similar studies from diversified cultures, especially from the Eastern collectivistic cultures, are needed to establish the cathartic effects of profanity in a more scientific fashion.

## Figures and Tables

**Table 1:** Descriptive statistics (*N* = 253).

Variable	Items	*α*	M	SD	%	Range		Skewness	Kurtosis
						Potential	Actual		
Profanity	62	0.983	129.841	58.791	41.88	62-310	62-310	0.767	-0.526
Depression	14	0.917	32.090	10.684	57.30	14-56	14-56	0.206	-0.817
Anxiety	14	0.906	28.122	9.986	50.21	14-56	14-56	0.515	-0.394
Stress	14	0.923	33.474	11.525	59.77	14-56	14-56	-0.054	-1.051

*α* = Cronbach's alpha; M = mean; SD = standard deviation.

**Table 2:** Correlation of the understudied variables (*N* = 253).

	Depression	Anxiety	Stress	Age	Education
Profanity	-0.182^∗∗^	-0.161^∗^	-0.250^∗∗^	0.031	0.016
Depression		0.674^∗∗^	0.753^∗∗^	-0.192^∗∗^	-0.136^∗^
Anxiety			0.727^∗∗^	-0.129^∗^	-0.097
Stress				-0.205^∗∗^	-0.138^∗^
Age					0.502^∗∗^

^∗∗^Correlation is significant at the 0.01 level (2-tailed). ^∗^Correlation is significant at the 0.05 level (2-tailed).

**Table 3:** Differences in the levels of depression, anxiety, and stress based on lower and higher profaners (*N* = 253).

	Lower profaners (*n* = 154)		Higher profaners (*n* = 99)		*t*(251)	*p*	Cohen's *d*
	M	SD	M	SD			
Depression	33.487	10.406	29.919	10.801	2.622	0.009	0.338
Anxiety	29.175	10.492	26.485	8.953	2.106	0.036	—
Stress	35.169	11.311	30.838	11.415	2.961	0.003	0.381

**Table 4:** Gender-based differences (*N* = 253).

	Men (*n* = 98)		Women (*n* = 155)		*t*(251)	*p*	Cohen's *d*
	M	SD	M	SD			
Profanity	143.235	58.392	121.374	57.638	2.924	0.004	0.377
Depression	31.194	11.062	32.658	10.435	1.062	0.289	0.137
Anxiety	27.439	9.986	28.555	9.996	0.866	0.388	0.112
Stress	31.439	11.467	34.761	11.413	2.252	0.025	0.291

## Data Availability

Data associated with this paper can be presented on demand.
